# Fasting and Systemic Insulin Signaling Regulate Phosphorylation of Brain Proteins That Modulate Cell Morphology and Link to Neurological Disorders*[Fn FN2]

**DOI:** 10.1074/jbc.M115.668103

**Published:** 2015-10-23

**Authors:** Min Li, Chao Quan, Rachel Toth, David G. Campbell, Carol MacKintosh, Hong Yu Wang, Shuai Chen

**Affiliations:** From the ‡ State Key Laboratory of Pharmaceutical Biotechnology and Ministry of Education Key Laboratory of Model Animal for Disease Study, Model Animal Research Center, Nanjing University, Pukou District, Nanjing 210061, China,; the § Medical Research Council Protein Phosphorylation Unit, College of Life Sciences, University of Dundee, Dundee DD1 5EH, Scotland, United Kingdom, and; the ¶ Division of Cell and Developmental Biology, College of Life Sciences, University of Dundee, Dundee DD1 5EH, Scotland, United Kingdom

**Keywords:** brain, cytoskeleton, insulin resistance, neurodegeneration, protein phosphorylation, MARKs, fasting, srGAP3

## Abstract

Diabetes is strongly associated with cognitive decline, but the molecular reasons are unknown. We found that fasting and peripheral insulin promote phosphorylation and dephosphorylation, respectively, of specific residues on brain proteins including cytoskeletal regulators such as slit-robo GTPase-activating protein 3 (srGAP3) and microtubule affinity-regulating protein kinases (MARKs), in which deficiency or dysregulation is linked to neurological disorders. Fasting activates protein kinase A (PKA) but not PKB/Akt signaling in the brain, and PKA can phosphorylate the purified srGAP3. The phosphorylation of srGAP3 and MARKs were increased when PKA signaling was activated in primary neurons. Knockdown of PKA decreased the phosphorylation of srGAP3. Furthermore, WAVE1, a protein kinase A-anchoring protein, formed a complex with srGAP3 and PKA in the brain of fasted mice to facilitate the phosphorylation of srGAP3 by PKA. Although brain cells have insulin receptors, our findings are inconsistent with the down-regulation of phosphorylation of target proteins being mediated by insulin signaling within the brain. Rather, our findings infer that systemic insulin, through a yet unknown mechanism, inhibits PKA or protein kinase(s) with similar specificity and/or activates an unknown phosphatase in the brain. Ser^858^ of srGAP3 was identified as a key regulatory residue in which phosphorylation by PKA enhanced the GAP activity of srGAP3 toward its substrate, Rac1, in cells, thereby inhibiting the action of this GTPase in cytoskeletal regulation. Our findings reveal novel mechanisms linking peripheral insulin sensitivity with cytoskeletal remodeling in neurons, which may help to explain the association of diabetes with neurological disorders such as Alzheimer disease.

## Introduction

Insulin resistance is a hallmark of type II diabetes that comes with major complications, such as nephropathy, retinopathy, and heart disease. Moreover, recent studies have highlighted the strong linkage of insulin resistance with an increased risk of depression, cognitive decline, and Alzheimer disease (1, 2). Together diabetes and dementia are reaching epidemic proportions and have major social and financial consequences. However, the molecular basis of the association between diabetes and brain decline is unknown. There is a common perception that the complications of diabetes such as nephropathies, heart disease, and cognitive decline are secondary consequences of vascular problems caused by loss of glucose control (3). However, it seems logical that such disorders could also be more direct consequences of dysregulated insulin action within the damaged tissues (4, 5).

Although the brain as a whole is classically viewed as an insulin insensitive organ, the insulin receptor (IR)^5^ is expressed in brain regions including the olfactory bulb, hypothalamus, and hippocampus (6). The signaling components downstream of IR such as insulin receptor substrate-1 (IRS-1), PI 3-kinase, and protein kinase B (PKB, also known as Akt) are widely expressed in the brain (7). Insulin signaling in the brain is important in the control of food intake (8), hepatic gluconeogenesis (9), energy homeostasis, and reproductive endocrinology (10). Besides these effects on metabolism and reproduction, insulin in the brain also regulates circuit function and plasticity by controlling synapse density (11). In humans, intravenously administered insulin under euglycemic hyperinsulinemic conditions improves cognitive functions (12), and intranasally administered insulin similarly results in an improvement in learning and memory formation without affecting blood glucose levels (13). Moreover, the brain in Alzheimer disease (AD) displays a resistance to insulin that is associated with dysfunctional IRS-1 (14). The upstream IRS-1 serine kinases including glycogen synthase kinase 3 (GSK-3), IκB kinase (IKK), JNK, and mechanistic target of rapamycin (mTOR) are activated in the basal state, resulting in higher basal IRS-1 serine phosphorylation. The elevated basal IRS-1 serine phosphorylation consequently causes insulin resistance in the brain in AD through down-regulation of its tyrosine phosphorylation by the insulin receptor (14). However, genetic ablation of the IR in mouse brain causes insulin resistance in the central nervous system but does not impair learning and memory formation (15). Therefore, it remains unclear how insulin regulates cognitive performance.

In many organs, fasting triggers physiological changes opposite to those elicited by insulin. As in other organs, fasting can activate cAMP-dependent protein kinase A (PKA) in the brain (16); the latter is important for many aspects of brain functions through regulating various cellular activities such as gene transcription (17) and receptor trafficking (18). Given the importance of PKA in learning and memory (17), it was therefore intriguing to find out whether fasting/PKA signaling is connected with insulin response in the brain.

During experiments to define molecular changes caused by insulin in multiple organs *in vivo*, we found that fasting induced phosphorylation of a number of proteins in the brain, whereas peripheral administration of insulin after a fast triggered dephosphorylation of these proteins. We isolated these proteins through an IP-MS (immunoprecipitation coupled with mass spectrometry) approach. Among the identified proteins were cytoskeleton regulators, namely slit-robo GTPase-activating protein 3 (srGAP3); Arf-GAP with GTPase, ANK, and PH domain-containing protein 2 (AGAP2); and microtubule affinity-regulating protein kinases (MARKs). These data revealed a novel regulatory pathway by which fasting and peripheral insulin regulates brain functions and which may help explain some of the links between diabetes and neurological disorders.

## Experimental Procedures

### 

#### 

##### Materials

Recombinant human insulin was from Novo Nordisk (Bagsvaerd, Denmark), forskolin and H-89 from Selleckchem (Shanghai, China), and microcystin-LR from Enzo Life Sciences (Farmingdale, NY). Precast NuPAGE® bis-Tris gels were from Thermo Fisher Scientific and protein G-Sepharose and glutathione-Sepharose 4B from GE Healthcare. All other chemicals were from Sigma-Aldrich or Sangon Biotech (Shanghai, China).

##### Antibodies

Antibodies against the HA tag and MARK4 were raised in sheep by the Division of Signal Transduction Therapy (DSTT), University of Dundee (UK) as reported previously (19). The antibody against total srGAP3 was raised in sheep by the DSTT, University of Dundee, using the recombinant GST-srGAP3 proteins as immunogen. The site-specific antibody against phospho-Ser^858^ on srGAP3 was raised in sheep by the DSTT, University of Dundee, using the synthetic phosphopeptide (GRVRLR***pS***DGAAIP, residues 852–864 of human srGAP3, where bold italic ***pS*** represents phosphorylated Ser^858^) as immunogen and column-purified against the same phosphopeptide. FLAG antibody (catalogue No. F1804) and GAPDH antibody (G8795) were from Sigma-Aldrich. Flotillin1 (FLOT-1) antibody (sc-25506), Bcl2 antibody (sc-7382), Bax antibody (sc-7480), and PKA-Cα antibody (sc-903) were from Santa Cruz Biotechnology. PKA2β antibody (ab75993) was from Abcam. Antibodies that recognize phosphorylated Ser^473^ on PKB (catalogue No. 9271) and phosphorylated Ser^133^ on cAMP response element-binding protein (CREB; catalogue No. 9191), anti-PKB (catalogue No. 9272), anti-CREB (catalogue No. 9197), the phospho-Akt substrate (PAS) antibody (catalogue No. 9611), the pS/T-PKA substrate antibody (catalogue No. 9621), and immobilized PAS antibody (catalogue No. 9646) were from Cell Signaling Technology. The WAVE1 antibody (catalogue No. 07-037) and SVP38 antibody (catalogue No. MAB368) were from Millipore. The site-specific antibody recognizing phospho-Ser^157^ on VASP (catalogue No. 676604) was from Calbiochem.

##### Molecular Biology

The cDNA encoding human srGAP3 (NM_014850) was cloned into the vector pcDNA5-FRT/TO-HA for expression in mammalian cells. Residue numbering of srGAP3 was according to the srGAP3 protein encoded by this human cDNA. Point mutation of srGAP3 was carried out using standard procedures. The sequence contexts of mutated sites on srGAP3 are: GRVRLR**s**DGAAIP (Ser^858^ in lowercase bold), AMRRS**s**SSSTEMM (Ser^1029^ in lowercase bold), and AMRRSS**s**SSTEMM (Ser^1030^ in lowercase bold). The cDNA encoding human Rac1 (NM_006908.4) was cloned into the vector pcDNA5-FRT/TO-FLAG for expression in mammalian cells. The cDNA encoding the Pak1-PBD spanning Lys^67^ to Ala^150^ of human Pak1 was cloned into the pGEX6P vector for protein expression in *Escherichia coli*. All DNA constructs were sequenced either by the service managed by Nick Helps, University of Dundee, or by Life Technologies, Inc.

##### Mouse Husbandry and Procedures

Mouse husbandry and procedures were approved by the Ethics Committees at the University of Dundee and Nanjing University. C57Bl6 mice were housed with a light/dark cycle of 12 h and free access to food and water unless stated otherwise.

For insulin injection, mice deprived of food overnight (16 h) were anesthetized with sodium pentobarbital (90 mg/kg of body weight) and injected intraperitoneally with a bolus of insulin (150 milliunits of insulin/g of body weight). For glucose injection, mice deprived of food overnight (16 h) were injected intraperitoneally with a bolus of glucose (2 g of glucose/kg of body weight). Twenty minutes after injection with insulin or glucose, the mice were killed for tissue collection via cervical dislocation. For refeeding, mice were allowed free access to food for 90 min after food deprivation overnight (16 h) and were killed via cervical dislocation for tissue collection after refeeding.

##### Tissue Homogenization and Measurement of Protein Concentration

Mouse tissues were homogenized in lysis buffer using a Polytron homogenizer (Kinematica, Switzerland) and further lysed on ice for 30 min as described previously (20). After lysis, tissue debris was removed from tissue lysates through centrifugation, and the protein concentrations of tissue lysates were determined using Bradford reagent (Thermo Fisher Scientific).

##### Isolation of Primary Cortical Neurons and Cerebellar Granule Cells

Primary cortical neurons were isolated as described previously (21). Briefly, neonatal pups (P0–P1) were killed by decapitation, and the cerebral cortex was removed, finely diced, and digested with trypsin. Dissociated cells were washed six times in serum-free Neurobasal A medium before passing through a 70-μm cell strainer. Cortical neurons were then plated on poly-d-lysine-coated dishes.

Primary cerebellar granule cells were isolated as described previously (22). Briefly, neonatal pups (P4–P7) were killed by decapitation, and the cerebellar external granule layer was dissected out, finely diced, and digested with trypsin. Tissues were triturated into a single cell suspension with fire-polished pipettes and then allowed to gravity-precipitate. The single cell suspension was passed through a 70-μm cell strainer and plated on poly-d-lysine-coated dishes.

##### Cell Culture, Transfection, Stimulation, and Lysis

Primary cortical neurons were cultured in Neurobasal A medium containing 1% fetal bovine serum (FBS), 0.4 mm glutamine, and 2% B27 supplement. Primary cerebellar granule cells were cultured in Neurobasal A medium containing 1% FBS, 2 mm glutamine and 2% B27 supplement. HEK293 cells were cultured in DMEM containing 10% FBS. HEK293 cells were transfected via a polyethylenimine-mediated method as described previously (20). The siRNAs for knockdown of the PKA catalytic subunits were as described previously (23). For treatments with forskolin or insulin, cells were deprived of serum for 12 h (basal) and then stimulated as indicated with forskolin at 10 μm or insulin at 100 nm for 30 min. When H-89 was used, it was added to the cell cultures at 10 μm prior to stimulation with forskolin. Cells were then lysed in ice-cold lysis buffer as described previously (24).

##### Immunoprecipitation

Immunoprecipitation was carried out as described previously (24). Briefly, the indicated antibodies were coupled to protein G-Sepharose and used for incubation with cell or brain lysates overnight at 4 °C. Following incubation, unbound proteins were removed after centrifugation, and resins were washed to remove nonspecific binding proteins. Immunoprecipitates were then eluted from resins in SDS sample buffer for subsequent analysis.

##### Electrophoresis and Immunoblotting

Cell or tissue lysates or immunoprecipitates were separated electrophoretically on commercial or laboratory-made gels. After electrophoresis, proteins were immunoblotted onto nitrocellulose membranes that were further incubated with the indicated primary antibodies at 4 °C for 16 h. After being incubated with horseradish peroxidase-conjugated secondary antibodies, the chemiluminescent signals were detected using ECL® (GE Healthcare).

##### Mass Spectrometry

After SDS-PAGE, colloidal Coomassie-stained bands were excised, digested with trypsin, and analyzed by LC-MS on an LTQ-Orbitrap (Thermo Finnigan) mass spectrometer coupled to a Dionex 3000 nano liquid chromatography system as described previously (25). For peptide and protein identification, raw files were converted to peak lists in Mascot generic format (MGF) files using raw2msm v1.7 software (Matthias Mann), and MGF files were then searched using a Mascot 2.2 in-house server against the Swiss-Prot database. Only peptides with Mascot ion score over 25 were considered, and only proteins with at least two unique peptides were considered. For phosphopeptide analysis, only phosphopeptides with ion scores over 18 were considered. The individual MS/MS spectra for the phosphopeptide ions were quantified using Xcalibur 2.2 software.

##### In Vitro Phosphorylation

The recombinant GST-srGAP3 proteins were expressed in *E. coli*, purified using glutathione-Sepharose 4B (GE Healthcare), and phosphorylated by a catalytic subunit of PKA (V5161, Promega) *in vitro* at 30 °C for 30 min as described previously (20).

##### GAP Activity Measurement

The GAP activity of recombinant GST-srGAP3 was measured with Rac1 as substrate using the GAP assay kit (BK105, Cytoskeleton Inc.).

##### Measurement of Active Rac1 in Cell Lysates

For the Rac1 activation assay, cells were lysed in lysis buffer (50 mm HEPES/KOH, pH 7.4, 100 mm NaCl, 4 mm MgCl_2_, 1 mm DTT, 1% Nonidet P-40, 10% glycerol, 10 mm NaF, 1 mm Na_3_VO_4_, 1 mg/ml leupeptin, 1 mg/ml pepstatin, and 1 mg/ml aprotinin). Purified recombinant GST-Pak1-PBD was immobilized on glutathione-Sepharose beads and incubated with cell lysates at 4 °C for 1 h. After nonspecifically bound proteins were removed by three washes with lysis buffer, the Pak1-PBD-bound active Rac1 was eluted from resins in SDS sample buffer and measured via Western blot.

##### Statistical Analysis

Unless stated otherwise, the data were analyzed via Student's *t* test, and differences were considered statistically significant at *p* < 0.05.

## Results

### 

#### 

##### Intraperitoneal Injection of Insulin after a Fast Decreases Phosphorylation of Proteins in the Brain

**FIGURE 1.**
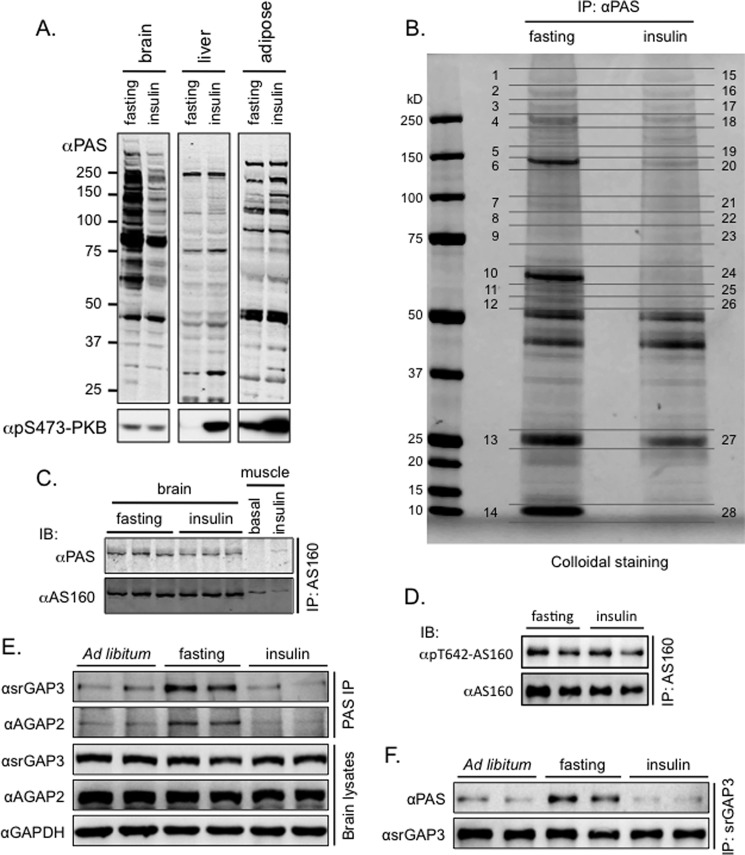
**Intraperitoneal injection of insulin triggers protein dephosphorylation in mouse brain.** *A*, PAS antibody-reactive phosphorylation and phosphorylation of Ser^473^ of PKB were detected in the lysates of brain, liver, and adipose tissue from mice subjected to overnight fasting (16 h) or to intraperitoneal insulin injection (20 min) after an overnight fast. *B*, PAS antibody-reactive phosphoproteins were immunoprecipitated (*IP*) using immobilized PAS antibody beads from brain lysates of mice subjected to overnight fasting (16 h) or to intraperitoneal insulin injection (20 min) after an overnight fast. The immunoprecipitated proteins, identified via mass spectrometry, included srGAP3, AGAP2, MARK3, and MARK4. *C* and *D*, the AS160 proteins were immunoprecipitated using the AS160 antibody from brain lysates (2 mg) and muscle lysates (0.2 mg) of mice subjected to overnight fasting (16 h) or to intraperitoneal insulin injection (20 min) after an overnight fast. PAS antibody-reactive phosphorylation (*C*) and Thr^642^ phosphorylation (*D*) of AS160 were detected in the immunoprecipitates using the PAS antibody and the phospho-Thr^642^-specific antibody, respectively. *IB*, immunoblot. *E*, PAS antibody-reactive phosphorylated proteins were immunoprecipitated using the immobilized PAS antibody beads from brain lysates of mice subjected to *ad libitum* feeding or overnight fasting (16 h) or to intraperitoneal insulin injection (20 min) after an overnight fast. The levels of srGAP3 and AGAP2 in the immunoprecipitates using the immobilized PAS antibody beads were detected using the specific antibodies recognizing srGAP3 and AGAP2, respectively. *F*, The srGAP3 proteins were immunoprecipitated using the srGAP3 antibody from brain lysates of mice subjected to *ad libitum* feeding or overnight fasting (16 h) or to intraperitoneal insulin injection (20 min) after an overnight fast. The PAS antibody-reactive phosphorylation of srGAP3 was detected in the immunoprecipitates using the PAS antibody.

We collected organs from mice that were injected intraperitoneally or not with insulin and interrogated the response to insulin by analyzing phospho-Ser^473^ of PKB and the phosphorylation of its potential downstream targets detected with the PAS antibody. As expected, the phosphorylation of PKB on Ser^473^ increased in the fat and liver in response to insulin (Fig. 1*A*). The phosphorylation of PKB on Ser^473^ was unchanged in the lysates of the brains from the mice that were intraperitoneally injected with insulin (Fig. 1*A*). By analyzing crude lysates, it became obvious that insulin stimulates the phosphorylation of many proteins in the fat and liver as detected with the PAS antibody (Fig. 1*A*). However, in striking contrast to these organs, the PAS-binding signals of many proteins were diminished in the crude lysates of the brains from the mice subjected to intraperitoneal injection of insulin (Fig. 1*A*).

These phosphoproteins were purified from mouse brain by immunoprecipitation with the PAS antibody, digested with trypsin, and identified using mass spectrometry (Fig. 1*B* and supplemental Table 1). Among the proteins identified, AS160/TBC1D4, GARNL1/RalGAPα1, and RalGAPα2 are known PAS-binding proteins and PKB substrates (20, 24, 26). Taking the Mascot score for each protein as a very approximate indicator of protein abundance, the scores for these proteins showed no decrease in the insulin-treated samples (supplemental Table 1). Furthermore, the PAS-binding signals and the phosphorylation of the major PAS recognition site (Thr^642^) were unaltered for the immunoprecipitated AS160 from the brains of the insulin-treated mice (Fig. 1, *C* and *D*). In contrast, a number of other proteins had Mascot scores that were markedly lower in the PAS-captured samples from the brains of insulin-treated mice than the scores from fasted animals (supplemental Table 1). Such proteins include regulators of cytoskeletal reorganization, namely MARKs, which are on the AMP-activated protein kinase branch of the human kinome and regulate microtubule dynamics; srGAP3, which is widely expressed in the brain and regulates the directionality of cell polarity, axon extension, and migration (27); and AGAP2, which is critical for the control of synaptic insertion of the AMPA receptor (28). The diminished levels of srGAP3 and AGAP2 in the PAS immunoprecipitates from the brains of the insulin-treated mice were further confirmed via an immunoblotting assay using the srGAP3- and AGAP2-specific antibodies, respectively (Fig. 1*E*). In a reciprocal experiment, the PAS-binding signals were markedly decreased on the srGAP3 immunoprecipitated from the brains of the insulin-treated mice (Fig. 1*F*). Because srGAP3 and MARKs have been implicated in certain neurological diseases (29, 30), we subsequently focused on the regulation of these proteins in this study.

##### Peripheral Insulin Sensitivity Links with Phosphorylation of Target Proteins in the Brain

**FIGURE 2.**
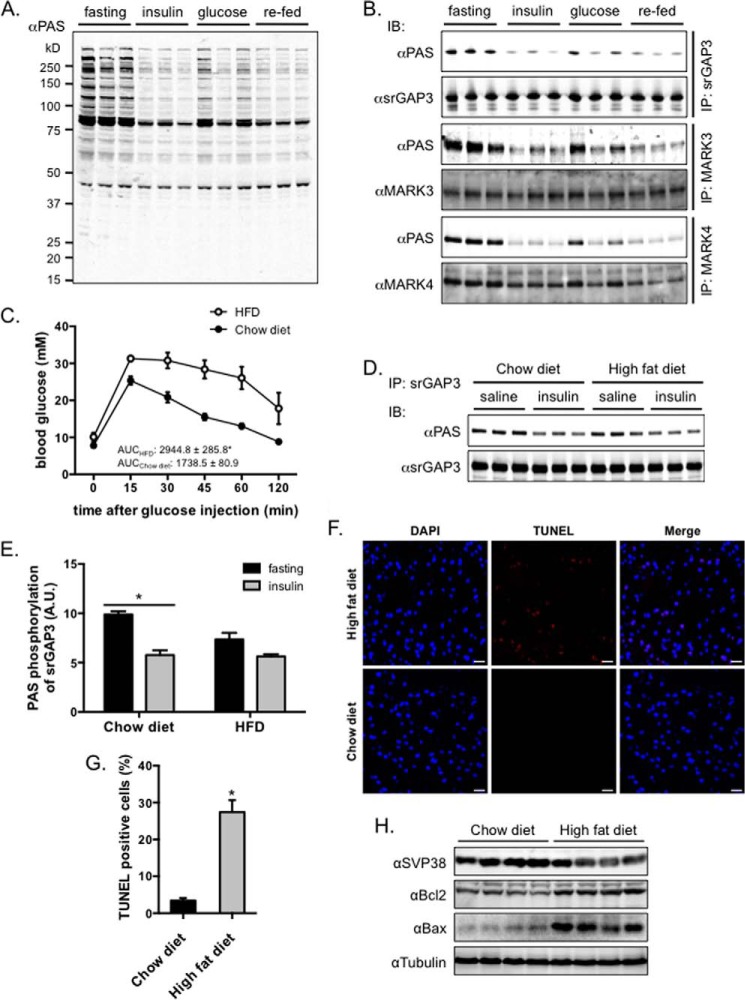
**Peripheral insulin sensitivity modulates phosphorylation of target proteins in mouse brain.** *A* and *B*, overnight fasted mice were injected intraperitoneally with insulin (20 min) or glucose (20 min) or allowed to refeed *ad libitum* for 90 min (three mice for each condition) before being culled for brain sampling. The PAS antibody-reactive phosphorylation was detected in brain lysates (*A*). The indicated proteins (srGAP3, MARK3, and MARK4) were immunoprecipitated from lysates, and their phosphorylation was detected using the PAS antibody (*B*). *C–E*, mice were fed on either a chow diet or high fat diet (*HFD*) for 5 weeks. The high fat diet induced insulin resistance in mice as evidenced by the glucose tolerance test (*n* = 5) (*C*). Overnight fasted mice were injected intraperitoneally with saline or insulin (20 min) before termination for brain sampling. The PAS antibody-reactive phosphorylation was detected on the srGAP3 proteins immunoprecipitated from brain lysates (*D*). The PAS antibody-reactive phosphorylation on the srGAP3 was then quantified (*n* = 3) (*E*). Statistical analysis was carried out using two-way analysis of variance. *IP*, immunoprecipitate; *IB*, immunoblot. *F* and *G*, TUNEL staining of brain sections of mice fed on chow diet or high fat diet for 16 weeks. *F*, representative TUNEL staining of brain sections. *G*, quantitation of TUNEL positive cells. The data were summarized from five brain sections for each condition. *H*, expression of SVP38, Bcl2, and Bax in homogenates of brains from mice fed on a chow diet or a high fat diet for 16 weeks. The data are given as the mean ± S.E.; *, *p* < 0.05.

Insulin injection is nonphysiological, and what we had observed in the brain could have been a secondary effect due to a fall in blood glucose or release of stress hormones, especially as these animals were fasted overnight. However, we suspect that insulin itself was the primary trigger because the PAS-binding signals in the crude brain lysates and on immunoprecipitated srGAP3, MARK3, and MARK4 from the brain lysates were also decreased when animals were injected with glucose or more physiologically allowed to refeed after a fast (Fig. 2, *A* and *B*). A common denominator for these conditions is increased blood insulin.

We next investigated whether insulin resistance in the peripheral tissues affected the phosphorylation/dephosphorylation of srGAP3 in the brains of mice subjected to intraperitoneal insulin injection after a fast. To this end, wild-type mice were fed a high fat diet for 5 weeks to induce insulin resistance. As expected, mice on the high fat diet displayed glucose intolerance (Fig. 2*C*), which indicated that they developed insulin resistance. Again, intraperitoneal injection of insulin decreased the PAS-binding signals on the srGAP3 immunoprecipitated from the brain lysates of control mice on a chow diet (Fig. 2, *D* and *E*). In contrast, intraperitoneal insulin injection caused a less prominent decrease in PAS binding to srGAP3 immunoprecipitated from mice on the high fat diet (Fig. 2, *D* and *E*). As reported previously (31), we found that the high fat diet induced apoptosis in mouse brain as evidenced by TUNEL staining (Fig. 2, *F* and *G*) and expression of apoptosis markers Bcl-2 and Bax (Fig. 2*H*). The expression of a synaptic vesicle marker, SVP38, was also decreased in the brain from high fat diet-fed mice (Fig. 2*H*).

##### Fasting Increases the Phosphorylation of the Target Proteins in the Brain

**FIGURE 3.**
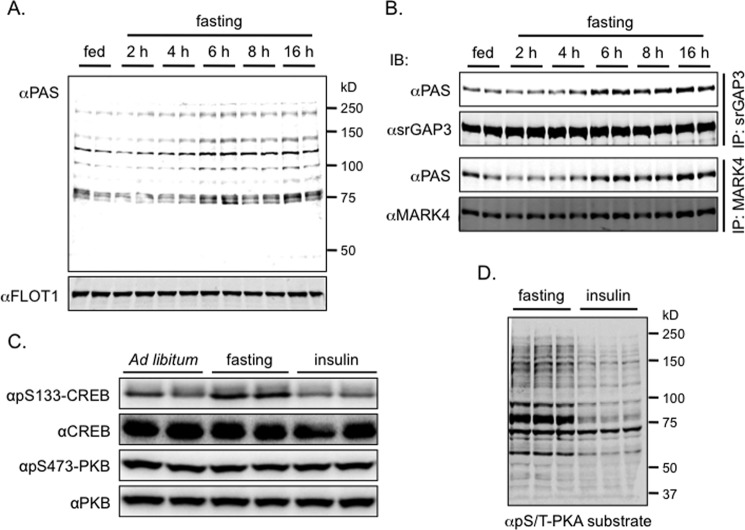
**Fasting induces protein phosphorylation in mouse brain.** *A* and *B*, mice were subjected to *ad libitum* feeding or deprived of food for the indicated time before termination for brain sampling. The PAS antibody-reactive phosphorylation was detected in brain lysates with FLOT-1 as a loading control (*A*). The indicated proteins (srGAP3 and MARK4) were immunoprecipitated from lysates, and their phosphorylation was detected using the PAS antibody (*B*). *C*, mice were subjected to *ad libitum* feeding or overnight fasting (16 h) or to intraperitoneal insulin injection (20 min) after an overnight fast before termination for brain sampling. Total and phosphorylation of CREB and PKB were detected in brain lysates. *D*, protein phosphorylation was detected using a pS/T-PKA substrate antibody in brain lysates of mice subjected to overnight fasting (16 h) or intraperitoneal insulin injection (20 min) after an overnight fast. *IB*, immunoblot.

We next investigated how fasting regulated the phosphorylation of the target proteins. To this end, mice were fasted for various periods before sampling. We found that the PAS-binding signals in the crude brain lysates and on immunoprecipitated srGAP3 and MARK4 from the brain lysates were all increased when the mice were fasted longer than 6 h (Fig. 3, *A* and *B*). The Ser^473^ phosphorylation of PKB was unchanged in the brains of fasted mice, suggesting that PKB was not responsible for the fasting-induced PAS-binding signals in the brain (Fig. 3*C*). It has been shown that fasting can activate PKA in the brain, which can increase phosphorylation of its substrate, CREB (16, 32). As reported previously, we found that the Ser^133^ phosphorylation of CREB was increased in the brains of fasted mice, and in contrast, intraperitoneal injection of insulin after a fast decreased the Ser^133^ phosphorylation of CREB in the lysates of mouse brains (Fig. 3*C*). When a generic phospho-Ser/Thr PKA substrate antibody was used to detect protein phosphorylation in mouse brains, a similar protein phosphorylation pattern as detected with the PAS antibody was found in the brains from fasted mice, and the phosphorylation signals were strongly decreased in the brains from insulin-injected mice (Fig. 3*D*). Together, these data suggest that PKA might be involved in the phosphorylation of the identified proteins such as srGAP3 and MARKs in the brains of fasted mice.

##### Forskolin Stimulates the Phosphorylation of srGAP3, MARK3, and MARK4 in Primary Neurons

**FIGURE 4.**
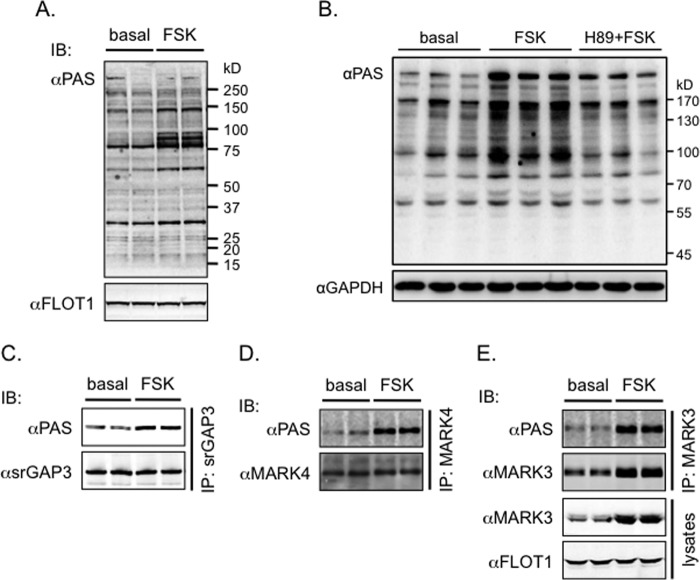
**Forskolin induces the PAS antibody-reactive protein phosphorylation in primary neurons.** *A*, primary cortical neurons were isolated from neonatal mice and subjected to stimulation with forskolin. The PAS antibody-reactive phosphorylation was detected in cell lysates with FLOT-1 as a loading control. *B*, primary cerebellar granule cells were isolated from neonatal mice and subjected to stimulation with forskolin in the presence or absence of H-89. The PAS antibody-reactive phosphorylation was detected in cell lysates with GAPDH as a loading control. *C–E*, the indicated proteins (srGAP3, MARK3, and MARK4) were immunoprecipitated from lysates of primary cortical neurons that were stimulated with or without forskolin, and their phosphorylation was detected using the PAS antibody. *IB*, immunoblot.

Forskolin, an adenyl cyclase activator that indirectly activates PKA through elevating cellular cAMP, was used to treat primary cortical neurons and cerebellar granule cells isolated from neonatal mice. The PAS-binding signals were markedly increased in the lysates of cortical neurons upon stimulation with forskolin (Fig. 4*A*) and presented a pattern similar to that observed in the brain lysates of fasted mice. Similarly, forskolin also increased the PAS-binding signals in the lysates of cerebellar granule cells, which was prevented by pretreatment with the nonspecific PKA inhibitor H-89 (Fig. 4*B*). The PAS-binding signals were increased on the immunoprecipitated srGAP3 and MARK4 from the lysates of cortical neurons treated with forskolin (Fig. 4, *C* and *D*). Forskolin also markedly elevated the MARK3 protein levels in cortical neurons, which parallels the PAS-binding signals on the immunoprecipitated MARK3 proteins (Fig. 4*E*).

##### Identification of Ser^858^ as a Regulatory Phosphorylation Site on srGAP3

**FIGURE 5.**
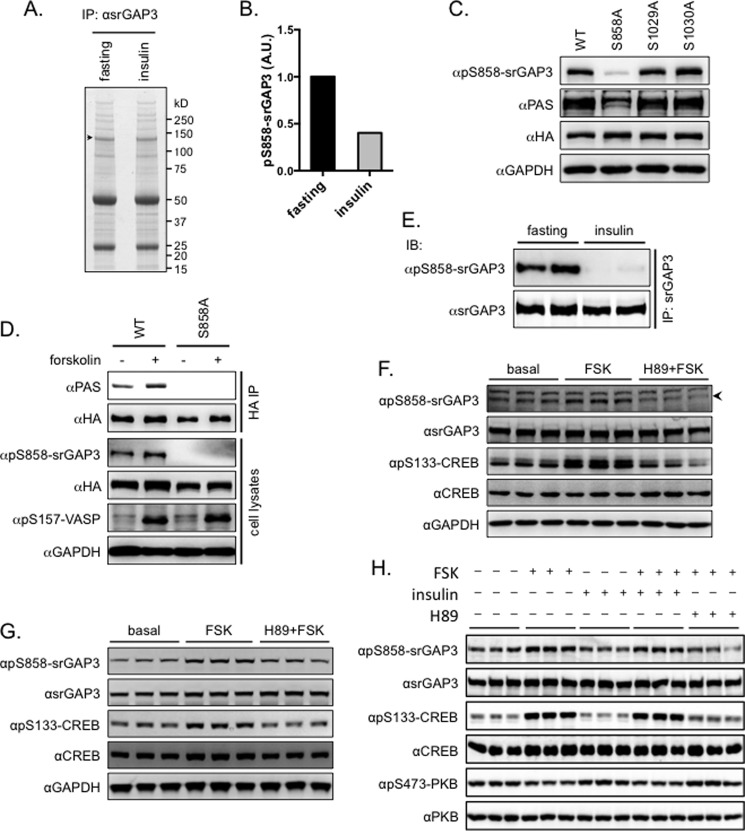
**Phosphorylation of the srGAP3 induced by fasting in mouse brain and forkolin in primary neurons.** *A*, the srGAP3 proteins were immunoprecipitated (*IP*) from brain lysates and subjected to phosphomapping analysis via mass spectrometry. *A.U.*, arbitrary unit. *B*, the srGAP3 proteins were immunoprecipitated from the brains of mice that were either fasted overnight (16 h) or intraperitoneally injected with insulin for 20 min after an overnight fast. Phosphorylated Ser^858^ was detected and quantified by measuring the area detected by the relevant extracted ion chromatograms on the immunoprecipitated srGAP3 proteins. The quantified intensities under fasting conditions were set to one, and the intensities under insulin injection conditions were normalized against the respective values under fasting conditions. The values shown are the mean of two experiments. *C*, The HA-tagged wild-type and mutant srGAP3 proteins were expressed in HEK293 cells. Phosphorylation of the HA-tagged srGAP3 proteins was detected using the PAS antibody and pS858-srGAP3 phospho-specific antibody. Total HA-tagged srGAP3 was detected using the anti-HA antibody with GAPDH as a loading control. The surrounding sequence for Ser^1029^ and Ser^1030^ is as follows: RRS**ss**SSTEMM (Ser^1029^ and Ser^1030^ shown in lowercase bold letters). *D*, HEK293 cells expressing the HA-tagged wild-type and mutant srGAP3 proteins were stimulated with or without forskolin. Phosphorylation of the HA-tagged srGAP3 proteins was detected using the PAS antibody and pS858-srGAP3 phospho-specific antibody. Phosphorylated VASP was detected using the pS157-VASP phospho-specific antibody with GAPDH as a loading control. *E*, the srGAP3 proteins were immunoprecipitated using the srGAP3 antibody from brain lysates of mice subjected to overnight fasting (16 h) or to intraperitoneal insulin injection (20 min) after an overnight fast. The Ser^858^ phosphorylation of srGAP3 was detected in the immunoprecipitates using the pS858-srGAP3 phospho-specific antibody. *F* and *G*, primary cortical neurons (*F*) and cerebellar granule cells (*G*) were isolated from neonatal mice and subjected to stimulation with forskolin (*FSK*) in the presence or absence of H-89. The Ser^858^ phosphorylation of srGAP3 was detected in cell lysates using the pS858-srGAP3 phospho-specific antibody. Total and phosphorylated CREB were detected in cell lysates with GAPDH as a loading control. The *arrowhead* in *F* indicates the signals for Ser^858^ phosphorylation of srGAP3. *H*, primary cerebellar granule cells were isolated from neonatal mice and subjected to stimulation with forskolin, insulin, or both. Total and phosphorylated PKB, srGAP3, and CREB were detected in cell lysates. *IP*, immunoprecipitate; *IB*, immunoblot.

We next carried out a detailed analysis of the phosphorylation of srGAP3 in mouse brain. To this end, the srGAP3 proteins were immunoprecipitated from the brains of the mice fasted overnight or injected with insulin after a fast (Fig. 5*A*), and phosphopeptides were identified on the precipitated proteins via mass spectrometry. Fourteen phosphopeptides were identified on the immunoprecipitated srGAP3 from mouse brain lysates (Table 1). The phosphorylated residue could only be precisely assigned for LRSDGAAIPR (Ser^858^ shown underlined), which displayed an over 2-fold decrease in ion intensity when the mice were injected intraperitoneally with insulin (Fig. 5*B*). The phospho-Ser^858^ and its surrounding sequence confer the recognition motif R*X*R*XX*p(S/T) of the PAS antibody. When Ser^858^ was substituted by a non-phosphorylatable alanine, the PAS-binding signals on the srGAP3 were diminished (Fig. 5*C*), suggesting that phospho-Ser^858^ is a primary binding site on the srGAP3 for the PAS antibody. To further study the phosphorylation of Ser^858^ on the srGAP3, we raised a site-specific phosphoantibody for phospho-Ser^858^. The specificity of the phospho-Ser^858^ antibody was confirmed by (phospho)peptide dot blots (data not shown) and immunoblot with the S858A mutant protein (Fig. 5*C*). The PAS-binding signals and phospho-Ser^858^ signals were both increased on the wild-type srGAP3 isolated from transfected cells stimulated with forskolin, but the response to forskolin was diminished on the srGAP3^S858A^ mutant protein (Fig. 5*D*). Using the phospho-Ser^858^-specific antibody, we further confirmed that Ser^858^ phosphorylation was decreased in the brain lysates of the mice that were injected intraperitoneally with insulin (Fig. 5*E*). In the primary cortical neurons and cerebellar granule cells, forskolin increased the phosphorylation of Ser^858^ on srGAP3, whereas this forskolin-induced Ser^858^ phosphorylation was prevented when cells were pretreated with the nonspecific PKA inhibitor H-89 (Fig. 5, *F* and *G*). In contrast, insulin could neither activate PKB, as evidenced by the Ser^473^ phosphorylation on PKB, nor inhibit the forskolin-stimulated Ser^858^ phosphorylation on srGAP3 in the lysates of treated cortical neurons (Fig. 5*H*), suggesting that down-regulation of phosphorylation of srGAP3 in the brain by systemic insulin administration (Fig. 5*E*) is not a direct effect of insulin in neurons and requires a secondary mediator that may derive from peripheral tissues.

**TABLE 1:** **Phosphopeptides identified on the srGAP3 in mouse brain** The srGAP3 proteins were immunoprecipitated from lysates of mouse brains, and phosphopeptides were detected on the immunoprecipitated srGAP3 proteins via mass spectrometry. The known modified residue is underlined. NA, not applicable (indicating that phosphorylated residues are present but cannot be assigned precisely).

Peptide name	P site
R.LRSDGAAIPR.R + p(S/T)	Ser^858^
R.SSSSSTEMMTTFKPALSAR.L + p(S/T)	NA
R.RSSSSSTEMMTTFKPALSAR.L + p(S/T)	NA
R.SSSSSSSGVGSPAVTPTEK.M + 2 p(S/T)	NA
R.MATFGSAGSINYPDKK.A + p(S/T)	NA
R.AAACPSSPHKIPLSR.G + p(S/T)	NA
R.SSSSSSSGVGSPAVTPTEK.M + p(S/T)	NA
R.STESIKSAASETYMSK.I + 2 p(S/T)	NA
R.STESIKSAASETYMSK.I + p(S/T)	NA
R.SGGDTHSPPRGLGPSIDTPPR.A + 2 p(S/T)	NA
K.NDLQSPTEHISDYGFGGVMGR.V + p(S/T)	NA
K.ASSKNDLQSPTEHISDYGFGGVMGR.V + p(S/T)	NA
R.SGGDTHSPPRGLGPSIDTPPR.A + p(S/T)	NA
R.HSSLGDHKSLEAEALAEDIEK.T + 2 p(S/T)	NA

##### PKA Forms a Complex with srGAP3 and WAVE1 and Phosphorylates srGAP3

**FIGURE 6.**
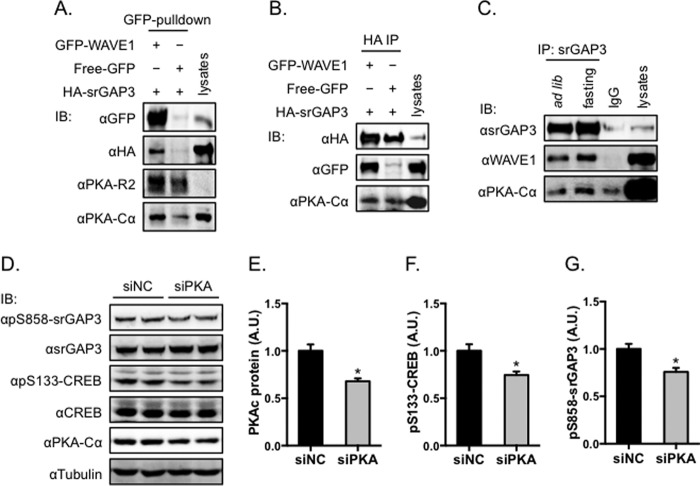
**PKA forms a protein complex with srGAP3 and WAVE1 and phosphorylates srGAP3 in cells.** *A* and *B*, HA-srGAP3 and GFP-WAVE1 were co-expressed in HEK293 cells. *A*, GFP-WAVE1 was immunoprecipitated from cell lysates, and HA-srGAP3, the endogenous PKA catalytic subunit, and the regulatory subunit were detected in the immunoprecipitates. *B*, in a reciprocal experiment, HA-srGAP3 was immunoprecipitated from cell lysates, and GFP-WAVE1 and the endogenous PKA catalytic subunit were detected in the immunoprecipitates. *C*, endogenous srGAP3 was immunoprecipitated from homogenates of brains of mice subjected to either *ad libitum* (*ad lib*) or overnight fasting (16 h). Endogenous WAVE1 and the PKA catalytic subunit were detected in the immunoprecipitates. *D–G*, Ser^858^ phosphorylation of srGAP3 upon knockdown of PKA. PKA catalytic subunit was knocked down via siRNA in HEK293 cells expressing HA-srGAP3. Phosphorylation and expression of HA-srGAP3, CREB, and PKA were determined via Western blot using tubulin as a loading control. *D*, representative immunoblots (*IB*). *E–G*, quantitative data, *n* = 5. The data are given as the mean ± S.E.; *, *p* < 0.05. *IP*, immunoprecipitate; *A.U.*, arbitrary unit; *siNC*, negative control siRNA.

It has been reported that the protein kinase A-anchoring protein (AKAP) WAVE1 is a srGAP3-interacting protein (33). Consistent with this report, we found that HA-srGAP3 could be co-immunoprecipitated with GFP-WAVE1 when these two proteins were co-expressed in cells (Fig. 6, *A* and *B*). Substantial amount of endogenous PKA could be detected in the immunoprecipitates (Fig. 6, *A* and *B*). When endogenous srGAP3 was immunoprecipitated from brain homogenates, endogenous WAVE1 and PKA could be detected in the immunoprecipitates (Fig. 6*C*). Importantly, the amounts of co-precipitated WAVE1 and PKA were substantially increased in the immunoprecipitates of srGAP3 from homogenates of fasted mice (Fig. 6*C*), which correlates with the increased Ser^858^ phosphorylation of srGAP3 (Fig. 5*E*). These data suggest that formation of the srGAP3-WAVE1-PKA complex may facilitate the phosphorylation of srGAP3 by PKA. To obtain more direct evidence that PKA phosphorylates srGAP3, we knocked down PKA via siRNA in cells and found that Ser^858^ phosphorylation of srGAP3 was significantly decreased in parallel with a lower level of phosphorylation of CREB upon PKA knockdown (Fig. 6, *D–G*). Together, these data show that PKA is an upstream kinase that can phosphorylate Ser^858^ of srGAP3.

##### Phosphorylation of srGAP3 by PKA Increased Its GAP Activity toward Rac1

**FIGURE 7.**
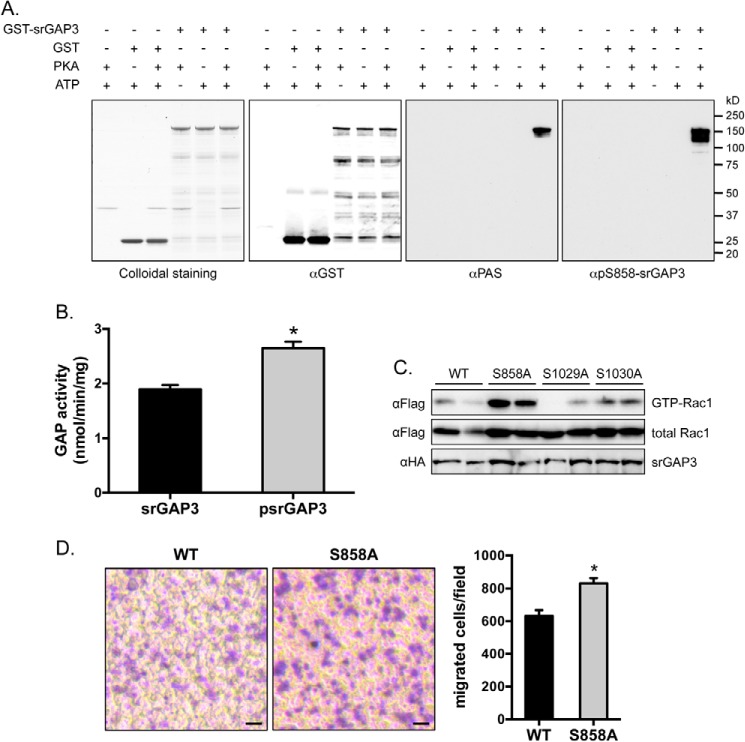
**Ser^858^ phosphorylation of srGAP3 increases its GAP activity toward Rac1.** *A*, GST-srGAP3 proteins were phosphorylated by PKA *in vitro*. The phosphorylation of GST-srGAP3 was detected using the PAS antibody or pS858-srGAP3 phospho-specific antibody. *B*, GST-srGAP3 proteins were phosphorylated by PKA *in vitro*, and their GAP activities toward Rac1 was determined. *n* = 3. The data are given as the mean ± S.E.; *, *p* < 0.05. *C*, the HA-tagged wild-type and mutant srGAP3 proteins were co-expressed with the FLAG-tagged Rac1 in HEK293 cells. The GTP-Rac1 was determined via immunoblotting after being isolated from cell lysates using recombinant Pak1-RBD. *D*, the wild-type and mutant srGAP3 proteins were expressed in DU145 human prostate cancer cells, and cell migration was measured using Corning Transwell inserts. At the end of the Transwell assay, the migrated cells were fixed, stained with crystal violet, and counted (*n* = 9). The data are given as the mean ± S.E.; *, *p* < 0.05.

To further study how PKA-mediated phosphorylation regulates srGAP3, we carried out an *in vitro* phosphorylation of bacterially expressed and purified GST-srGAP3 fusion proteins using a PKA catalytic subunit. The *in vitro* phosphorylated srGAP3 proteins could be detected using the PAS antibody as well as the phospho-Ser^858^ antibody (Fig. 7*A*).

Consistent with a previous report (29), the full-length srGAP3 recombinant protein displayed GAP activity toward Rac1 (Fig. 7*B*). Interestingly, the *in vitro* phosphorylation of the srGAP3 by PKA significantly increased its GAP activity toward Rac1 (Fig. 7*B*). When the srGAP3 and Rac1 were co-expressed in HEK293 cells, the srGAP3^S858A^ mutant protein increased the levels of the GTP-bound active form of Rac1 as compared with the wild-type srGAP3, srGAP3^S1029A^, and srGAP3^S1030A^ mutant proteins (Fig. 7*C*), further suggesting that Ser^858^ phosphorylation increases the GAP activity of srGAP3. Consistent with its effect on Rac1 activation, expression of the srGAP3^S858A^ mutant protein in DU145 cells enhanced cell migration as compared with the wild-type srGAP3 protein (Fig. 7*D*).

## Discussion

In this study, we found that fasting induced phosphorylation of a set of proteins in mouse brain, whereas peripheral administration of insulin down-regulated their phosphorylation in the brain. The data presented here identify a novel regulatory mechanism linking fasting and insulin to signal transduction in the brain, which may help to elucidate the molecular basis of the association between diabetes and brain decline in neurodegenerative diseases.

Over the last few years, brain insulin signaling has been a focus of research aimed at unraveling the molecular mechanisms that underlie the association of insulin sensitivity with brain cognitive functions. Cumulative evidence indicates that insulin resistance is associated with cognitive decline. For instance, insulin response was markedly decreased in post-mortem hippocampal formation of AD cases as compared with normal cases (14). The levels of a number of insulin signaling molecules including IR, IRS-1, and PKB were significantly reduced in the post-mortem frontal cortex of individuals with both AD and type II diabetes (34). However, brain insulin resistance caused by deletion of IR in mouse brain did not affect learning and memory formation (15). It has been proposed that compensatory mechanisms may operate in the absence of IR to prevent brain decline (35). An alternative hypothesis is that peripheral insulin resistance contributes to cognitive impairment in neurodegeneration, enhanced by factors such as vascular lesions and the generation of neurotoxic lipids (36). Our results show that peripheral injection of insulin down-regulates the phosphorylation of a set of proteins in the brain, whereas insulin treatment of primary neurons does not alter the phosphorylation of these proteins, which is in line with this latter hypothesis. Our findings raise key questions about the nature of the mediator (if there is such) that transduces the peripheral insulin signaling into the brain, which phosphatase is activated or which kinase is inhibited in the brain upon peripheral injection of insulin to mediate the down-regulation of phosphorylation of the target proteins, and whether this decrease in protein phosphorylation regulates cognitive functions and, if so, how.

Although we have established that acute fasting induces the phosphorylation of a set of target brain proteins, the identity of the relevant kinase(s) *in vivo* remains unclear; however, several lines of evidence suggest PKA might be the one. In primary neurons, the PKA-activating agent forskolin increases phosphorylation of a similar set of target proteins in which the overall pattern detected by the PAS antibody is similar to the one in the brain induced by acute fasting, indicating that PKA might be the responsible kinase in the brain. The *in vitro* phosphorylation of srGAP3 by PKA and the decreased phosphorylation of srGAP3 upon knockdown of PKA in cells are also consistent with this kinase being a candidate for phosphorylation of the target proteins in the brain. In cells, AKAPs interact with both PKA and its substrates, bringing them into proximity for PKA to phosphorylate its substrates (37). It has been shown that the srGAP3 interacts with WAVE1, a known AKAP (33, 38). Our findings that fasting induces the formation of an srGAP3-WAVE1-PKA complex in mouse brain further indicates that PKA could be the kinase that phosphorylates Ser^858^ of srGAP3 in fasted animals. The activation of PKA involves the cAMP-stimulated dissociation of the regulatory subunit of PKA from its catalytic subunit (39), which makes it difficult to measure PKA activation directly in the brain. However, the phosphorylation of CREB, a known PKA substrate, indicates that PKA was activated in the brain by acute fasting as reported previously (32). The phosphorylation of CREB is proposed to mediate the antidepressant-like effects of acute fasting (32). It will be interesting to investigate whether the phosphoproteins discovered in this study also contribute to the antidepressant-like effects of acute fasting.

Cytoskeleton dynamics play an essential role in maintaining brain functions, and dysregulations of cytoskeletal proteins are linked to neurodegenerative diseases (40). The MARKs can phosphorylate the microtubule-associated proteins MAP4, MAP2c, and Tau on their microtubule-binding domains, which consequently detach these microtubule-associated proteins from microtubules and increase the dynamic instability of microtubules (41, 42). In primary rat hippocampal neurons, overexpression of MARK4 causes Tau hyperphosphorylation and results in synaptic toxicity (43). Elevated MARK4 expression and MARK4-Tau interactions were found in post-mortem human AD brains, further highlighting the importance of this kinase in AD (44). The MARKs are activated via phosphorylation of the threonine residue in the activation loop by upstream kinases including LKB1 (19) and MARKK/TAOK1 (45). In contrast, glycogen synthase kinase 3 (GSK-3) inhibits MARK2 by phosphorylating Ser^212^ of this kinase (46). Therefore, it will be critical to carry out a detailed study in the future to address how the phosphorylation of the MARKs detected by the PAS antibody regulates their activities and whether their deregulation contributes to AD or other tauopathies. A further cytoskeleton regulator identified in this study is srGAP3, which influences cytoskeleton dynamics through the down-regulation of Rac1 signaling (27, 47, 48). Loss of the srGAP3 in humans has been linked to mental retardation (29), and deletion of the srGAP3 in mice impairs learning and memory by affecting dendritic spine formation (49, 50). Therefore, our discoveries suggest that fasting and systemic insulin signaling regulates brain cytoskeleton dynamics and brain functions through controlling the phosphorylation of the MARKs and srGAP3.

Taken together, our findings reveal a novel regulatory mechanism that links fasting and systemic insulin sensitivity to brain functions. In the longer term, dissecting the underlying mechanism more precisely may identify therapeutic targets and provide “biomarkers” to track aspects of diabetes other than glucose-related effects, so that damaging changes can be detected early.

## Author Contributions

M. L., C. Q., R. T., D. G. C., H. Y. W., and S. C. performed experiments, analyzed data, and reviewed the manuscript. C. M. contributed to the experimental design and writing of the manuscript. H. Y. W. and S. C. designed the experiments and wrote the manuscript. All authors approved the final paper.

## Supplementary Material

Supplemental Data

## Footnotes

5 The abbreviations used are:

IR: insulin receptor
AD: Alzheimer disease
PAS: phospho-Akt substrate
AS160: 160-kDa Akt substrate
srGAP: slit-robo GTPase-activating protein
AGAP2: Arf-GAP with GTPase, ANK, and PH domain-containing protein 2
MARK: microtubule affinity-regulating protein kinase
PI: phosphoinositide
AKAP: protein kinase A-anchoring protein
CREB: cAMP response element-binding protein
bis-Tris: 2-[bis(2-hydroxyethyl)amino]-2-(hydroxymethyl)propane-1,3-diol
DSTT: Division of Signal Transduction Therapy (University of Dundee)
VASP: vasodilator-stimulated phosphoprotein.
