# Supplementary material for: Fasting and Systemic Insulin Signaling Regulate Phosphorylation of Brain Proteins That Modulate Cell Morphology and Link to Neurological Disorders
Source: J Biol Chem. 2015 Oct 23;290(50):30030–41. doi: 10.1074/jbc.M115.668103 (PMC4705965; doi:10.1074/jbc.M115.668103)
Supplement: Supplemental Data [file supp_290_50_30030__index.html]

Fasting and systemic insulin signaling regulate phosphorylation of brain proteins that modulate cell morphology and link to neurological disorders — Fasting and Systemic Insulin Signaling Regulate Phosphorylation of Brain Proteins That Modulate Cell Morphology and Link to Neurological Disorders — Insulin Sensitivity and Brain Protein Phosphorylation — Supplemental Data 

# Fasting and Systemic Insulin Signaling Regulate Phosphorylation of Brain Proteins That Modulate Cell Morphology and Link to Neurological Disorders

## Supplemental Data

- Supplemental Data 1 (.docx, 17 KB) - Supplemental Table 1 Caption
- Supplemental Data 2 (.xlsx, 40 KB) - Supplemental Table 1
