# Supplementary material for: Fasting and Systemic Insulin Signaling Regulate Phosphorylation of Brain Proteins That Modulate Cell Morphology and Link to Neurological Disorders
Source: J Biol Chem. 2015 Oct 23;290(50):30030–41. doi: 10.1074/jbc.M115.668103 (PMC4705965; doi:10.1074/jbc.M115.668103)
Supplement: Supplemental Data [file 10.1074_M115.668103_jbc.M115.668103-2.docx]

**Supplemental Table 1 Protein identification in the immunoprecipitates from mouse brain lysates**

Phosphorylated proteins were immunoprecipitated using the PAS antibody from brain lysates of the mice that were either fasted overnight (16 h) or intraperitoneally injected with insulin for 20 min after an overnight fast. The immunoprecipitated proteins were separated on SDS-PAGE (see Fig 1B), and the bands were excised, digested with trypsin and identified via mass-spectrometry.
